# Electrification of Transit Buses in the United States Reduces Greenhouse Gas Emissions

**DOI:** 10.1021/acs.est.2c07296

**Published:** 2024-02-19

**Authors:** Sofia S. Martinez, Constantine Samaras

**Affiliations:** † Civil and Environmental Engineering, Carnegie Mellon University, 5000 Forbes Avenue, Pittsburgh, Pennsylvania 15213, United States; ‡ Wilton E. Scott Institute for Energy Innovation, Carnegie Mellon University, 5000 Forbes Avenue, Pittsburgh, Pennsylvania 15213, United States

**Keywords:** public transit, electrification, buses, emissions, fleet replacement, decarbonization, United States

## Abstract

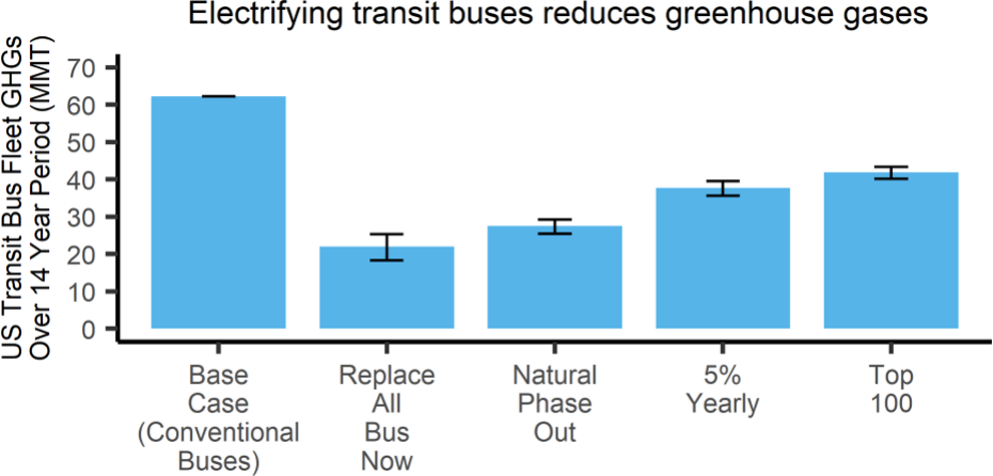

The transportation sector is the largest emitter of greenhouse gas emissions (GHGs) in the United States. Increased use of public transit and electrification of public transit could help reduce these emissions. The electrification of public transit systems could also reduce air pollutant emissions in densely populated areas, where air pollution disproportionally burdens vulnerable communities with high health impacts and associated social costs. We analyze the life cycle emissions of transit buses powered by electricity, diesel, gasoline, and compressed natural gas and model GHGs and air pollutants mitigated for a transition to a fully electric U.S. public transit bus fleet using transit agency-level data. The electrification of the U.S. bus fleet would reduce several conventional air pollutants and has the potential to reduce transit bus GHGs by 33–65% within the next 14 years depending on how quickly the transition is made and how quickly the electricity grid decarbonizes. A levelized cost of driving analysis shows that with falling capital costs and an increase in annual passenger-kilometers of battery electric buses, the technology could reach levelized cost parity with diesel buses when electric bus capital costs fall below about $670 000 per bus.

## Abstract 2

This paper quantifies the life cycle emissions mitigated by electrifying the entire U.S. public transit bus fleet over a range of scenarios and estimates the levelized costs.

## 1 Introduction

The United States (U.S.) transportation sector accounted for 28% of the total U.S. energy use and 67% of the total U.S. petroleum use in 2021.^1^ The transportation sector is also the largest emitter of greenhouse gases (GHGs), accounting for 28% of the approximately 6.3 gross billion tons of carbon dioxide equivalent (CO_2–e_) emitted in 2021.^2^ Hence, the deep decarbonization of transportation is critical to achieving U.S. climate committments. GHGs from passenger vehicles represent approximately 58% of the transportation sector GHGs, while trucks and aircraft represent about 23 and 9%, respectively.^1^ A considerable shift to public transit could reduce GHGs, helping to expand low-carbon mobility options during the transition to electric mobility.^3,4^ Yet, to maximize potential GHG reductions, a considerable scaling-up of electrified public transit vehicles, service, infrastructure, and ridership would be required.

Public transit agencies have a crucial role in providing mobility and access to the public and reducing energy use and pollution.^5^ In the U.S., many communities are still underserved by transit, and the lack of access to transportation has been shown to have a negative effect on the health of those facing this transportation barrier, especially in marginalized and vulnerable communities.^6,7^ The California Air Resources Board lists diesel particulate matter as a toxic air contaminant due to the relationship between diesel emissions and adverse health effects, including lung cancer.^8^ Emissions from high traffic areas have been shown to affect people of lower socioeconomic status and marginalized groups at higher rates in California, and electric public transportation can help mitigate the health effects while keeping members of these communities connected to resources.^9,10^ In the U.S., marginalized communities are disproportionately burdened by PM_2.5_ pollution, experiencing 56–63% excess pollution relative to their consumption.^11^

The interest in zero-tailpipe emissions battery electric buses (BEBs) is rising, and the major transit agencies are beginning to make electrification commitments. For example, New York City and Los Angeles have both committed to making their bus fleet fully electric within the coming decades, and the state of California has also committed to transition by 2040.^12^ In 2021, the U.S. passed the Infrastructure Investment and Jobs Act, commonly known as the Bipartisan Infrastructure Law, which included $5.6 billion in funding for low or no emission transit buses.^13^

While there is interest in the transition to BEBs, many agencies face barriers that prevent them from electrifying their transit bus fleet. Some barriers to electrification include high upfront capital costs for BEBs, costs of infrastructure upgrades and installations, local electrical grid issues, and other challenges.^14^ Even BEBs with large battery packs can vary in achievable daily driving range due to other factors, such as road grade, occupancy, driver aggression levels, use of other technology on board (such as air conditioning), and weather.^15^ Furthermore, agencies switching to electric buses face uncertainties and technology risks, which could require revision of major portions of their current system and routing to enable opportunity charging.^16,17^ This could require additional resources, and some agencies are still rebounding from the effects of COVID and other pressures.^18^

We evaluate the costs, life cycle emissions avoided, and timeline of transitioning the entire U.S. public transit bus fleet to BEBs using agency-level data gathered by the U.S. Federal Transit Administration (FTA). Most existing literature has focused on analyzing city-scale transitions.^19−21^ In a national analysis, Holland et al. estimated the pollution damages and net present value for electrifying the U.S. bus fleet at the county level;^22^ see a literature review in Supporting Information 1.1. Our paper adds to the literature by using transit agency-level data on existing transit fleet age, uses life cycle impacts, and presents a set of future transition scenarios to compare impacts. Because of the decline in battery prices and updated future projections about changes in the electricity system, our analysis adds new context for decision-makers.

## 2 Materials and Methods

### 2.1 Using the FTA Data Sets

We acquired data on transit buses operated by U.S. transit agencies from the U.S. Federal Transit Administration (FTA) National Transit Database (NTD) for 2021^23^ (see data cleaning details in Supporting Information 1.2 and specific NTD data sets in Supporting Information 2.1). We began our analysis by estimating how many miles current fossil-fueled buses drive on their daily routes. Then, the sizes of the battery packs were estimated using the FTA’s Bus Research and Testing Center’s test results of vehicle battery efficiency. Finally, we developed bus replacement schedules based on the remaining years prior to eligible replacement in the current fleet of transit buses across the U.S.

### 2.2 Fleet Age Exploration

**Figure 1:**
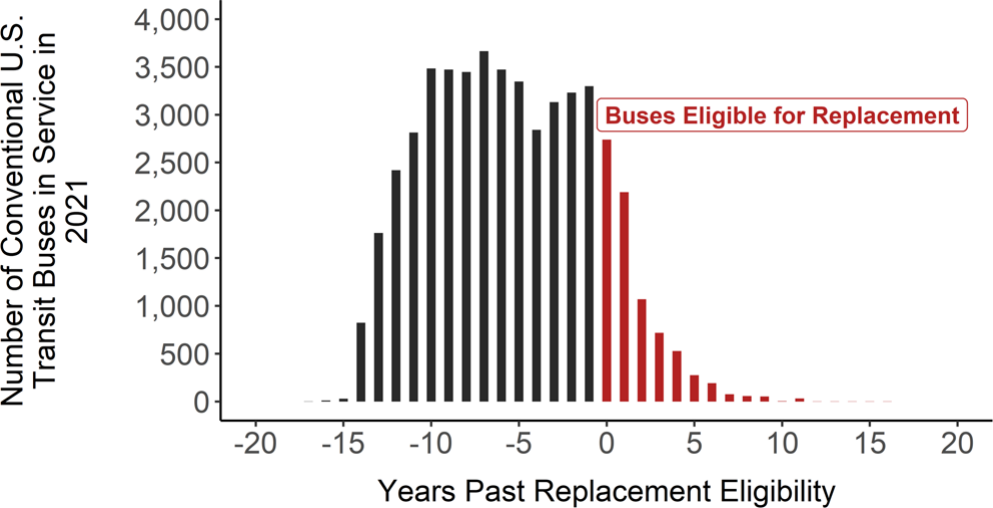
Distribution of years past replacement eligibility in the U.S. transit bus fleet, with positive values indicating existing buses eligible for replacement as of 2021. Data derived from (FTA, 2021).

The useful life benchmark of most transit buses is 12 years, the minimum standard set by the FTA, after which they can be phased out of the transit fleet, while some buses remain in service longer.^24^ Transit agencies list their own useful life benchmark in the data, and some agencies list their BEBs’ useful life benchmark up to 25 years. Taking manufacture year (Figure S1), rebuilds, and listed useful life benchmarks into account, we show in Figure 1 how many of the U.S. transit buses are eligible for replacement based on agencies’ own reported useful life benchmark. The buses in the negative range of Figure 1 are eligible for replacement in the years after 2021. About 22% of the U.S. bus fleet was already eligible for replacement in 2021, which may defer expenses on new capital costs, but because older buses typically run less efficiently, increase fuel costs, and produce more GHGs and air pollutants than newer buses. Similar figures for New York’s MTA, Chicago Transit Authority, and Los Angeles County MTA can be found in Supporting Information section 1.3 (Figures S4–S9).

### 2.3 GHG Emissions and Air Pollutants

We considered four sources of GHGs and air pollutants. The first is the direct tailpipe emissions from buses of differing fossil fuel types along with the particulate matter from tire and brake wear of all bus types (tank-to-wheels emissions). The second source is emissions generated from manufacturing the batteries that power BEBs. The third source of emissions is from conventional fuel production (well-to-tank emissions). The final source of emissions considered is electricity production to charge BEBs.

All considered GHGs (CO_2_, CH_4_, and N_2_O) are converted to CO_2_e using the global warming potential values from the Intergovernmental Panel on Climate Change (IPCC) in the Fifth Assessment Report (AR5).^25^ The air pollutants considered are CO, SO*_x_*, NO*_x_*, PM_10_, PM_2.5_, and VOCs. The tailpipe, tire, and brake wear emissions will be collectively called mobile emissions in this paper. The mobile emissions are gathered from the California Air Resources Board (CARB) Emission Factors model (EMFAC).^26^ CARB gathers mobile emission data on several specific transportation types, including urban buses with data for the four dominant fuel types in the U.S. transit bus fleet: gasoline, diesel, natural gas, and electricity. Electric buses are included because they emit particulate matter (PM) through tire and brake wear. Due to the added weight of the batteries and their management systems in BEBs, they are likely to emit PM through tire wear at a greater rate than tire wear from ICE vehicles.^27^ The data we gathered from the EMFAC model were the 2023 urban bus data for all available model years measured from a sample of buses in the state of California, but we generalized the emissions other than electricity emissions to the entire U.S. transit bus fleet (Supporting Information 1.4). After a comparison with our FTA data, missing data points were identified and estimated (Supporting Information 1.5). For a discussion on limitations of this data, see Section Supporting Information 1.9. The data for the emission of tailpipe GHGs and air pollutants are from the Argonne National Laboratory’s Greenhouse gases, Regulated Emissions, and Energy use in Technologies (GREET) model.^28^ The GREET model is divided into two cycles. The fuel cycle calculates the emissions from the use of different fuel types, including emissions generated from extracting, refining, and transporting these fuels (well-to-wheel). These emission factors have been gathered and used in the following analysis for low-sulfur diesel, gasoline, and compressed natural gas (Supporting Information 2.3 for data).

The vehicle cycle of the GREET model calculates the emissions generated from raw material extraction, vehicle component manufacturing, and vehicle assembly (cradle-to-gate emissions).^28^ This vehicle cycle provides an estimate of emissions generated by the battery manufacturing component of BEBs (Supporting Information 2.3 for data).^29^ The rest of the vehicle components are assumed to be equivalent to those of conventional buses. Ten models of BEBs were identified in the data set that also had specifications available. These specifications included cathode chemistry, battery pack size ranges, and efficiencies (kWh/mile). Three battery chemistries were identified: NMC (51%), LFP (19%), and LTO (9%). The rest (21%) were assigned to be the most common BEB model, which had an NMC battery.

Efficiency parameters were applied to account for transmission, distribution, and battery charging losses. According to the U.S. Energy Information Administration, the average efficiency of transmission and distribution in the U.S. is about 95%.^30^ An NREL evaluation of a BEB in Golden, Colorado, found an average charging efficiency for inductive charging and plug-ins to be about 85–90%.^31^ The value of 85% was chosen for this analysis.

Furthermore, BEB batteries may need replacements before the vehicle itself reaches its end of life.^21^ Battery replacement depends on the chemistry type, conditions, and proper use and care to ensure minimal battery degradation. A recent study has shown that degradation can be optimized with charging rates to maximize cost savings.^32^ Our analysis accounts for all BEBs needing one battery replacement in their lifetime and assumes a battery SOC from 10 to 80% as full battery depletion. The sensitivity analysis includes a range of potential battery replacements from 0 to 2.

Electricity production emission data were gathered from the 2020 non-baseload subregional EPA eGRID data (Supporting Information 2.3).^33^ We chose non-baseload data because any produced electricity-charging BEBs in the near term will be an added load to marginal grid demands. This is an important distinction because as more electricity is demanded, marginal power plants will need to serve this demand. Projecting into the future, we model electricity becoming cleaner over time by assuming that the emissions decrease through 2035 due to existing U.S. policy represented by the Inflation Reduction Act reaching levels 50–79% lower than the current values of CO_2_ and between 42–94% lower for NO*_x_* and SO*_x_*.^34^ To map eGrid electricity regions to the transit bus data for each agency, we used the EPA Power Profiler^35^ along with the transit agency address.

While our scenarios project a future of decarbonization for the electricity grid, we have not accounted for a future of emission reductions within the mining and battery assembly sectors due to the industry’s distribution across several countries. This is a conservative upper bound assumption for the battery sector, and actual emissions are likely to be even lower, especially as battery supply chains grow in the U.S.

### 2.4 Estimating Required BEB Range

**Figure 2:**
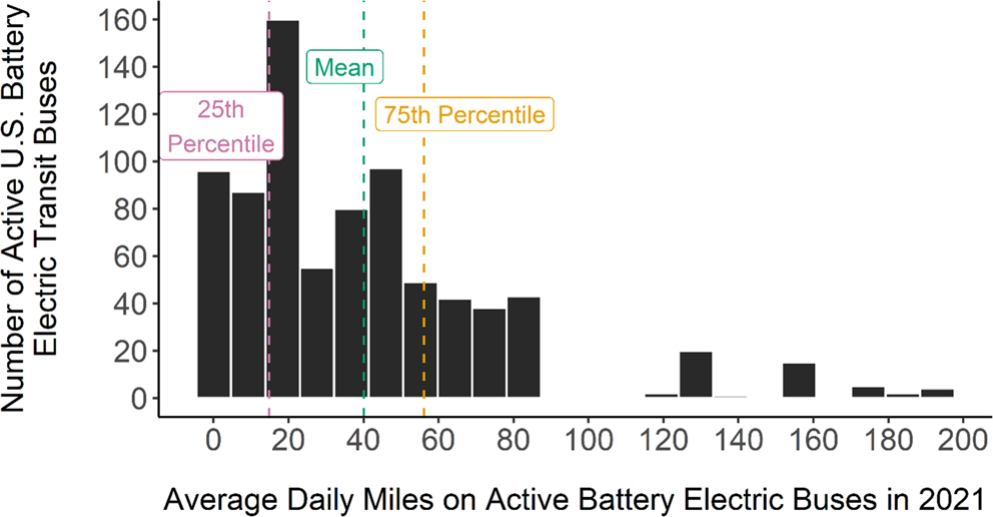
Average daily miles of U.S. electric transit buses in active service will be in use in 2021. The data have a mean of about 38 miles a day but with a wide distribution. There are four agencies currently operating BEBs at an average daily mileage greater than 100 miles.

To estimate a daily mile range for BEBs that will replace the fossil fuel fleet, we examine the average daily mileage of the BEBs currently in service, as seen in Figure 2. It is important to note that this daily mile range includes any opportunity charging agencies achieve between routes. The range of daily miles traveled by BEBs in service is considerably smaller than the range of fossil fuel buses (Figure S2). This is because the refueling time for electric vehicles in general is a lot longer than conventional vehicles. Due to this, the BEBs that travel more in a day than the battery range enabled by a single charge require opportunity charging or depot charging during time otherwise spent in revenue service. The mean of the distribution in Figure 2 is approximately 40 miles, the 25th percentile is about 15 miles, and the 75th percentile is about 56 miles. Comparatively, more than 55% of the fossil fuel fleet has a daily range smaller than 100 miles, and almost 99% of the fleet has a daily mile range under 200 miles. After informal discussions with several transit agencies, it was revealed that some agencies were testing BEB models for only a short time. Some agencies may purchase a vehicle from a manufacturer and test it for a month, and if the vehicle did not perform as promised, it was returned. Events such as these could have a large impact on the distribution of the reported actual range data. Other agencies mentioned that they had completed this limited testing and received only their full order of BEBs recently. This also has the potential to skew the reported data toward a lower daily range than a true measure since the NTD data do not require agencies to list how many months or days each individual vehicle was used in the year. As agencies become more familiar with the technology, more observations are included, and as battery technology progresses, we expect these reported ranges to trend toward higher values.

### 2.5 Battery Electric Bus Pricing

To obtain battery prices, the American Public Transportation Association’s (APTA) Public Transportation Vehicle Database was used.^36^ This database is smaller than the FTA’s National Transit Database but includes data about confirmed orders and potential orders of service vehicles in the future, including estimated prices. While these prices are subject to adjustments, these estimates are likely to be more representative of current BEB prices than those of previous years. Filtering this data set for BEBs of a manufacture year of 2021 yields 44 vehicles with a price range of $618 000–$952 000. The mean price of these BEBs is approximately $745 000.

To estimate the price of the buses in the future, the Argonne National Laboratory (ANL) Benefit Analysis (BEAN) model was used.^37^ This model was chosen over others due to the ANL accounting for the increase in price per kWh of a battery used in a heavy-duty vehicle, such as a transit bus. The battery price of this model starts at $302/kWh in 2021 and decreases to a range of $150–$80/kWh in 2035 depending on the level of technological advancement. The model itself only provides prices for the years 2021, 2027, 2035, and 2050; so, to estimate the price in every year omitted, a decay function was fitted to the three price points from 2021 to 2035. Figure S12 shows the equation used and the result of this extrapolation (Supporting Information 2.2).

### 2.6 Scenario Methodology

We developed several scenarios in this analysis of how the U.S. bus fleet could fully transition to BEBs. These scenarios can be used to provide options for stakeholders considering this transition. To calculate the emissions for battery production in these scenarios, we use the daily driving ranges of the buses and their energy consumption in kWh/mi from the FTA’s Larson Transportation Institute’s Bus Research and Testing Center testing reports.^38^ In all cases but the base case, we use a replacement procedure that assigns conventional buses a BEB model that conforms to their daily driving needs and the number of seats they can offer their riders. The batteries are sized to be able to make the daily trip needed while staying in an acceptable SOC and recharging at a depot at the end of the day. All replacement BEBs were assigned NMC battery replacements since these buses had the lowest estimate of energy consumption. Buses that seat more than 60 passengers were given 2 replacement buses since the models we were considering were all smaller (Supporting Information 1.6). We do this to capture the heterogeneity of the transit bus fleet, including bus sizes and daily driving demands. Except for scenario 1, the portion of the fleet that was not set to reach its replacement within 14 years of 2021 was calculated as driving the full 14 years as a conventional ICE bus in terms of tailpipe emissions.

As discussed, this analysis considers the future of declining emissions from electricity generation. We consider the current non-baseload emission rates used in 2021 and decline linearly until 2035. The 2035 electricity emissions are based on the percentage reductions from 2021 to 2035 as projected by Bistline et al.^34^ in response to current policies. The authors provide a range of possibilities that have been included in the sensitivity analysis of this paper (Supporting Information 1.7).

#### 2.6.1 Scenario 0: Base Case

For a comparison between the scenarios and a business-as-usual case, we estimate how much emissions would be generated by using the 2021 fleet as-is starting in 2021 for the next 14 years. We do not assume any changes in the tailpipe emissions or annual VMT.

#### 2.6.2 Scenario 1: Immediate Bus Replacement (Replace All Bus Now)

This first transition scenario considered is an analysis that assumes that all ICE buses in the U.S. transit fleet will be replaced immediately. The purpose of such an analysis is to provide a high-level overview of an immediate replacement. In other words, it serves the purpose of establishing a theoretical limit: an immediate replacement is unfeasible due to supply and manufacturing time. Even if all buses were immediately purchased, it is likely that it would take years to generate a sufficient supply and assemble the vehicles.

#### 2.6.3 Scenario 2: Natural Transition (Natural Phase Out)

As previously seen in Figure 1, we can estimate based on useful life benchmarks when specific agencies’ fleets are able to be replaced. According to this previous figure, around 3–4 thousand buses will need to be replaced in each of the coming years. Scenario 2 will show the emissions of the current buses being used until their useful life benchmark has been reached, in which case they will be replaced by BEBs in the 14-year emission analysis.

#### 2.6.4 Scenario 3: 5% Yearly Transition

Scenario 3 is a more gradual transition scenario, where only a smaller percentage of the fleet is replaced every year until the fleet is fully electric. In this scenario, the fleet will be transitioned to BEBs by about 5% every year, beginning with the buses that are already past their useful lives. In this transition method, the pace of the required manufacturing ramp-up is slower.

#### 2.6.5 Scenario 4: Replace Largest 100 Agencies

The fifth scenario is a replacement of only the largest 100 bus fleets by the agency. In this scenario, we use a similar method to scenario 3 except only the largest 100 agencies are transitioned to BEBs and the others are still included in the emission scenario, but they are counted as having similar technology throughout the 14-year analysis timeline.

## 3 Results and Discussion

### 3.1 Scenario 1: Immediate Bus Replacement

Replacing the entire fossil fuel powered U.S. bus fleet immediately using the method outlined in Supporting Information 1.6 would cost about $39.5 billion.

### 3.2 Scenario 2: Natural Transition (Natural Phase Out)

Because an estimated 22% of the U.S. fleet is already eligible for replacement, the cost to replace all of these was added to the first year. Accounting for these buses, there is an initial cost of replacement of nearly $8.5 billion. After this initial cost, the replacement costs per year vary from around $2.0 to $3.1 billion until 2035.

### 3.3 Scenario 3: 5% Yearly Transition

To replace 5% of the fleet each year until the entire 2021 fleet is turned over would cost on average $3.1 billion per year, and the transition would not be completed until 2045.

### 3.4 Scenario 4: Replace Largest 100 Agencies

The cost of this scenario replacement is $30.9 billion. This is approximately 81% of the sum of the total transition in scenario 3. Also compared to this scenario, about 81% of the fleet belonged to the largest 100 agencies and were modeled as being transitioned in this scenario.

### 3.5 Overall Scenario Emission Comparison

**Figure 3:**
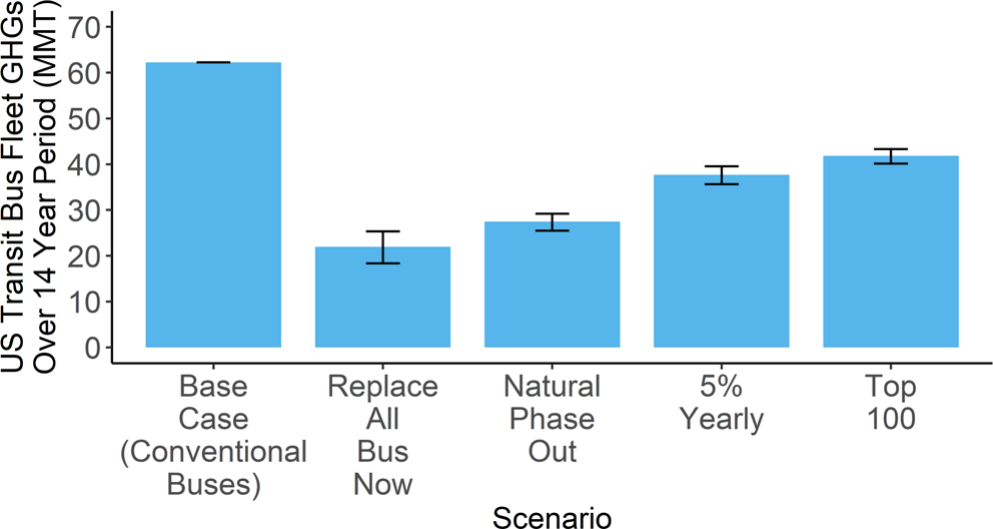
Scenario comparison of GHGs (measured in millions of tons) for the U.S. public transit bus fleet transitioning to BEBs, generated over a 14-year analysis timeline. In these scenarios, the GHG emissions of the electricity grid are declining linearly from 2021 until a grid 65% cleaner is achieved in 2035. The greatest variability comes from considering the extent of this decline in emissions of the electricity grid. Bistline et al. considered a range from 50 to 79%. In all cases, the results show that electrification is beneficial. Parameters used in the high and low estimates can be found in Table S2.

**Figure 4:**
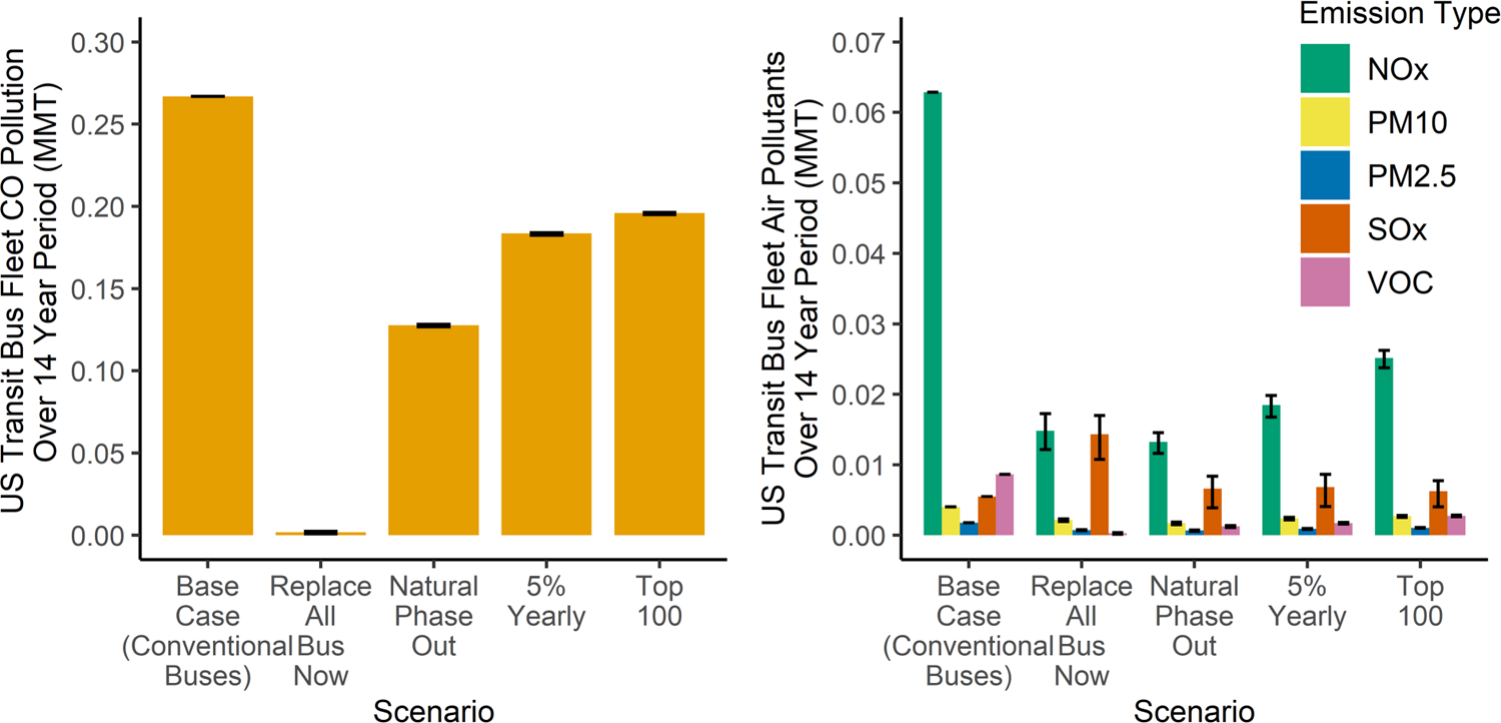
Scenario comparison of air pollutant emissions for transitioning to BEBs (measured in million metric tons). The left side shows the emission of CO on a different scale from the rest of the emissions shown on the right. The only pollutant to increase under these scenarios is SO*_x_*, which is mostly associated with coal-fired electricity generation, which continues to decline as a share of electricity generation. Bistline et al. project that NO*_x_* emissions from electricity will decrease by 62% (range of 42–82%) and SO*_x_* emissions will decrease by 73% (range of 49–94%) by 2035. The transition scenarios of Natural PhaseOut, 5% Yearly, and Top 100 show that if SO_*x*_ emissions are able to decrease quickly, there can still be some SO*_x_* mitigation over the 14-year analysis period. As the electricity and industrial systems transition to net-zero emissions, SO*_x_* emissions will continue to decline. Understanding the local changes and impacts of SO*_x_* emissions under rapid electrification and decarbonization across power, transport, and industry is an important topic for future research.

Figure 3 shows a comparison of GHGs generated by each scenario over the 14-year analysis period. In these scenarios, the potential GHGs mitigated vary from 65% or about 40 million metric tons (MMT) in scenario 1 (Replace All Bus Now) to 33% or about 20 MMT in scenario 4 (Top 100). Scenario 2 (Natural Phase Out) mitigates about 35 MMT of GHGs, a 56% reduction from the base case, and Scenario 3 (5% Yearly) mitigates about 25 MMT for a 39% reduction. Figure 4 shows that the results follow a similar pattern for air pollutants. Our base case calculation of life cycle CO_2_-e per passenger mile results in a value of 0.81 lbs per passenger mile (0.23 kg per passenger-km). This is 27% more than the FTA’s estimation from 2010 of 0.64 lbs per passenger mile (0.18 kg per passenger-km).^4^

The base case scenario where no transition to electrification occurs generated more emissions than any transition to BEBs. The emission savings from a rapid transition in the U.S. bus fleet are shown in scenario 1. While scenarios 2 and 3 are more realistic in terms of manufacturers being able to produce the BEBs necessary, they also show that the longer the transition to BEB takes, the less reduction in GHGs and air pollutants realized over time. Even if the electric grid decarbonization progressed slowly, bus electrification still reduced GHGs in all of our scenarios. For scenarios and emissions where very little variability exists, this may be due to most of these emissions coming from the conventional buses only. Most of our variation focuses on the different quantity and sizes of BEB batteries needed to replace the existing fleet, but the existing fleet’s schedule is only varied between scenarios. Thus, the largest variation of this analysis exists between scenarios and depends on the schedule of the fleet’s transition to BEBs. See Figure S11 to see the yearly emission reduction for each pollutant.

### 3.6 Levelized Cost of Driving

The levelized cost of driving (LCOD) calculation compares the cost of technology between different fuel types by combining the impact of capital costs, maintenance costs, infrastructure costs, fuel efficiency, and fuel prices into a single metric.^45^ These values are for simple comparison and do not account for any other costs, such as registration, unforeseen repairs, etc. The following equation and definitions are adapted from NREL.^45^
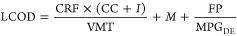
1

The capital recovery factor (CRF) is calculated as
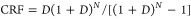
2where *D* is the discount rate and *N* is the lifetime of the vehicles in years. CC and *I* are capital costs and charging infrastructure costs, respectively. *M* is the annual vehicle maintenance costs and VMT is the annual vehicle miles traveled. FP is the price of the fuel for the technology being evaluated in dollars per gallon of diesel equivalent (DGE). Finally, MPG_DE_ is the mileage of the technology being evaluated in miles per gallon of diesel equivalent. Table 1 shows a summary of the inputs and assumptions for the LCOD analysis.

**Table 1:** Levelized Cost of Driving Values

parameter	diesel bus	compressed natural gas bus	electric bus with rapid charging	sources
capital cost (2023$)	557 000	649 000	745 000	(21,23,39)
infrastructure ($/bus)	0	62 000	56 000	(21)
maintenance ($/mi)	1.83	1.83	1.58	(21)
annual vehicle miles traveled	27 005			median value of entire data set
fuel price ($/DGE)	4.58	3.68	4.82	(40−42) all from January 2023.
MPDGE	4.7	5.2	22.0	(23,28)
discount rate (%)	5			(43)
eligible replacement (years)	12			(44)

**Figure 5:**
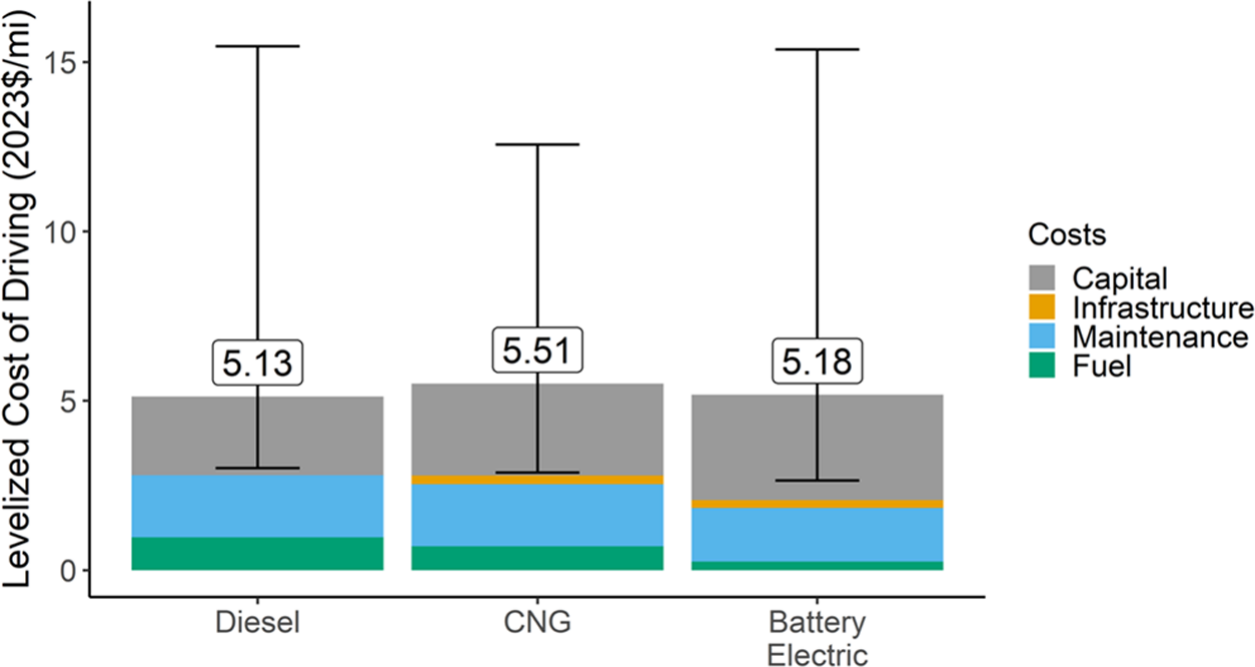
Levelized cost of driving analysis comparison among three fuel types. The sum of all noncapital costs is cheaper for the BEBs than the conventional buses. The high variability of the analysis comes from a wide range of feasible annual VMT. A sensitivity analysis can be found in Supporting Information 1.8.

All dollar values shown are adjusted to a 2023-dollar value using the consumer price index.^46^ The inflated prices of capital costs and infrastructure costs are rounded to the nearest thousand dollars. The results shown in Figure 5 are divided into their cost categories.

The price of electricity was adjusted by the efficiency of electric vehicle charging (85%) to account for the losses at the plug that agencies pay for but that does not end up in the battery packs.^31^ The conversions to diesel gallon equivalent (DGE) were made using fuel specifications listed in the GREET 2022 model.^28^

Overall, the levelized cost of driving BEBs is currently greater than that of diesel without considering external grant funding or the pricing of environmental externalities. As mentioned, Federal financial support exists for agencies to adopt no- or low-emission technologies. The FTA can provide 80% of capital costs in funding to agencies for vehicles that emit low or no tailpipe emissions.^47^ To make the electric technology cost-competitive with the dominant diesel technology, the capital costs of the BEBs would need to be around $670 000. Variation in local electricity tariffs and demand charges will affect the LCOD, and detailed local analysis is needed. The sensitivity analysis (Supporting Information 1.8) shows that annual mileage is the second most influential parameter in the analysis behind capital costs. These estimates provide a near-term price target for manufacturers and policymakers.

The levelized cost of driving analysis is meant to simply compare the cost of technologies at a high level; however, if other parameters were to be taken into account, such as the social cost of carbon or other health benefits, these could push BEB technology to cost parity or below. For example, if we apply a $51/metric ton value for the social cost of carbon (SCC) dioxide^48^ and consider that the transition to BEBs in the top 100 agencies scenario mitigates over 20 million metric tons of carbon dioxide over 14 years using 54 100 replacement BEBs (about 700 more than are currently used), then we can calculate the GHG cost savings per mile as
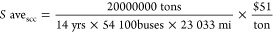


This yields a savings of almost $0.06/mi. This value brings the LCOD of the BEBs to a slightly lower price than for the other technologies. Recent literature has suggested that the true mean SCC is $185 per metric ton^49^ or higher. See Supporting Information 1.8 to see how the sensitivity of the other parameters affects the LCOD calculations.

The LCOD analysis shows that a critical component of making BEB technology affordable is its capital cost and driving range. Additional federal aid or a faster continued decline in BEB capital costs would help transit agencies replace their fleets at competitive costs.

Our analysis shows that the life cycle emissions from transitioning to battery electric buses charged by the electricity grid are less than those of the current bus fleet. Faster replacements are more capital intensive and would need a significant ramp-up in production capabilities for the U.S., but serve to demonstrate the maximum theoretical potential for emission reductions from the U.S. bus fleet in the next 14 years.

Also, by considering the levelized cost of driving and a realistic range for BEB replacement buses, we illustrate both the challenges and opportunities of transitioning to a BEB fleet. While the LCOD shows that BEBs are not yet always cost-competitive with diesel, we show that when taking into consideration the social cost of carbon BEB technology can be cheaper. These costs do not include the more immediate health savings from reducing the amount of particulate matter generated by diesel-powered buses that generally travel through more dense areas and affect more vulnerable populations. While we examined conventional buses in this paper, research has also shown that shared automated vehicles could improve equity in transit systems with reduced costs by acting as a complement to existing transit service to expand mobility and access. Continued advances in automated technology and assessment of equity issues in automation are necessary before transit agencies can begin widespread adoption of these vehicles.^50^

The U.S. is making commitments to bus electrification in the Bipartisan Infrastructure Law and the goals set by individual states and cities to reduce the impact of their own fleets.^13^ While there are still barriers facing BEBs in the market, our analysis shows that across a range of scenarios, the U.S. benefits from an earlier transition to an electric transit bus fleet through the mitigation of many health-damaging air pollutants and greenhouse gases.

## Supporting Information Available

Expanded literature review; additional methods; figures; and tables for transit agency data; emissions; and sensitivity analyses (PDF)
