# Supplementary material for: Electrification of Transit Buses in the United States Reduces Greenhouse Gas Emissions
Source: Environ Sci Technol. 2024 Feb 19;58(9):4137–44. doi: 10.1021/acs.est.2c07296 (PMC10919085; doi:10.1021/acs.est.2c07296)
Supplement: Supplementary file 1 — es2c07296_si_001.pdf [file es2c07296_si_001.pdf]

Supporting Information for

# Electrification of Transit Buses in the United States Reduces Greenhouse Gas Emissions

**Sofia S. Martinez and Constantine Samaras**

Civil and Environmental Engineering, Carnegie Mellon University, Pittsburgh, PA, United States of America

E-mail: csamaras@cmu.edu

Tables: 5

Figures: 12

Pages: 16

## SI 1 Notes

### *SI 1.1 Literature Review*

Public transit agencies have a crucial role in U.S. infrastructure in providing mobility and access to the public, reducing energy use and pollution, and enabling rapid transportation. Many communities are still underseved by transit, and lack of access to transportation has been shown to have a negative effect on the health of those facing this “transportation barrier”, especially in minoritized and vulnerable communities [1], [2]. The reduction and removal of these fine particles from highly populated areas would result in improved health for the these populations in addition to the reduced GHG emissions. One strategy to increase transportation mobility and access while reducing GHG and air pollutant emissions is to invest in cleaner technology, specifically electric buses charged by an electricity system that continues to get cleaner.

While the interest in zero-emission BEBs is rising, replacing the fleet with BEBs is a tailpipe solution. The GHG-intensity of electricity, while drastically improved since 2005, is not yet low-carbon in many regions of the country. In 2020, the U.S. produced 4.01 trillion kilowatt-hours of electricity and generated 1.55 billion metric tons of $CO_2$ [3]. Transit agencies cannot be emission-free until the source of the electricity is also emissions free. However, electricity generation has gotten cleaner in the U.S. [4], and the emissions profile of electric buses will continue to improve as the electric grid gets cleaner over time. Also, unlike electric passenger vehicles the federal funding towards BEBs is more less likely to have issues with equitable allocation [5].

BEBs face similar challenges to battery electric passenger vehicles such as their driving range is highly variable depending on the characteristics of the routes they travel and their locations and high capital costs. Due to these differences from conventional ICE buses, routes may need to be reconfigured to accommodate changes in range or to optimize the location of charging infrastructure [6]. The capital costs for these vehicles are currently higher than comparable diesel buses, but the cost of lithium-ion battery electric buses has declined by 89% since 2010 and is expected to drop another 50% by 2025 [7]. Options for addressing these upfront costs include leasing the entire bus, leasing only the battery, and leasing the charging depots [6]. These all allow for smaller upfront costs and combined can give agencies a window of testing the new equipment by minimizing risks.

In 2020, Quarels, Kockelman, and Mohamed did a life cycle cost analysis of replacing Austin, Texas’ 360-bus fleet with BEB’s at a rate of 30 new buses per year. The authors assumed a diesel bus cost of $300k and a BEB cost of $650k. While accounting for initial costs, maintenance costs, operation costs, fuel costs, and the price of new charging infrastructure over the course of the bus’s lifetime, they found that the BEB’s were not yet competitive with diesel buses [8]. In 2017, Tong, et al., modeled the costs and benefits of replacing Pittsburgh, Pennsylvania’s bus fleet to BEBs using the initial bus price, fuel costs, O&M costs, and upfront infrastructure costs. Their assumed bus prices were $485k-600k for diesel and $800k-1,200k for BEBs. The authors did consider the possibility of replacing the fleet using federal grants to help pay for the new BEBs (80% of the new bus price, which is the maximum the FTA will provide [9]). The authors found that the federal grants were necessary to make the BEBs competitive to diesel buses [10]. Lajunen’s cost-benefit analysis on BEBs used a model to compare the life cycle costs of the vehicles on routes with different parameters such as speed, acceleration, number of stops, duration, etc. These routes were located in Helsinki, Braunschweig, New York City, and Orange County, CA. The author concluded that on each route, the life cycle costs of BEBs were more than those of conventional diesel buses although not by a large amount. Considering that this paper was published in 2014 and battery technology is becoming more robust and less expensive, the life cycle costs of BEBs could be more competitive [11]. In 2021, Holland et al. estimated the pollution damages and net present value for electrifying the U.S. bus fleet at the county level. They find that electric buses have an average positive net present value of $8,000 per bus at 3% discount when compared to the direct expenses and secondary environmental damage expenses of diesel buses across the country [12].

There are two options for charging infrastructure for BEBs, overnight charging and en-route charging. The first, overnight charging, assumes that when a bus is parked overnight it will receive a full battery charge (usually over the course of many hours) and then slowly use this charge throughout the day. The second, en-route charging, assumes that as buses drive along their routes there will be small windows of opportunity at bus stops or breaks for charging that help to extend battery state of charge and vehicle range. This is generally also accompanied with overnight charging. Mohamed, et al., found that overnight charging didn’t require as large of a transformer to serve charging needs as an en-route (opportunity) charger which would save money on infrastructure costs. However, the

authors also found that overnight charging had a higher demand load factor which increases the cost of the electricity [13]. Tong, et al., reported BEB charging infrastructure as $45,000 per bus for fast chargers and $55,000 per bus for slow chargers [10]. The lower cost of fast charging could be due to the fact that fast chargers can be built to charge more buses at a time. This can be seen in Quarels et al. as they reported an overnight charger to cost $50,000 per unit while being able to service 2 buses at a time. They also reported en-route DC fast charges as being able to serve 20 buses simulataneoulsy and costing $110,000 [8].

### *SI 1.2 FTA Data Cleaning*

The 2,900 U.S. transit agencies that report data to the FTA combined served more than 3.9 billion passenger miles (6.4 billion km) in 2021. Nearly 48% of these miles are serviced by a bus, the majority by far. Second and third are demand response transit and heavy rail with 17% and 16% of the share of mileage, respectively. The remaining 19% is divided between other modes of transport such as commuter rail, vanpool, commuter bus, and light rail along with all other modes sharing less than a percentage. There are over 68,500 buses listed in the FTA's National Transit Database raw data set [14]. Of the 68,500 total transit buses in the U.S., 35,900 are diesel-powered, 14,800 are compressed natural gas, 9,700 are hybrid diesel, and 2,500 are gasoline. The remaining 5,600 buses are either of an unreported fuel type or an alternative fuel type (such as hydrogen or liquified petroleum gas). Any fuel type associated with on-vehicle combustion causes tailpipe GHG emissions. Of these alternative fuel types there are approximately 919 zero-emission battery electric buses (BEBs).

The FTA data comprises information about the agencies, including vehicles (buses, trains, trolleys, etc.), passenger miles served, vehicle miles traveled, daily range, energy usage, operating data, and more within the given year. The database is comprised of many datasets which are grouped by information type. The primary dataset used in this paper is the "2021 Revenue Vehicle Inventory" (SI 2.1 for access). These data are grouped by vehicle type within each agency. For example, Spokane Transit Authority in Spokane, WA has ten model year 2005 Gillig Low Floor buses and they have nineteen, 2006 Gillig Low Floor buses. This statement represents two entries to the dataset. Each entry may differ based on year, manufacturer, make, model, etc. This grouping is because agencies rarely purchase single buses at a time. These purchases are bought in batches so the new additions to the fleet are similar. The FTA NTD is presumed to be mostly complete because this is the national collection of transit data that is required to be reported by agencies every year by law [14]. There are small exceptions given to smaller agencies that do not require them to report all necessary data [15].

As mentioned, the NTD vehicle dataset contains information on vehicle types other than buses. The mode of interest in this study is "MB" which is a general field for buses. The original raw dataset for all vehicles included 33,689 rows of data. After this filter, there were 8,991 rows of data representing 68,592 vehicles. The vehicle types included in this subset were those other than what these researchers considered to be transit buses including vans, minivans, SUVs, and school buses along with some unnamed types. The vehicle types of interest for this study were articulated buses, buses, cutaways, and double decker buses. This filter removed 3,246 vehicles leaving 65,346 transit buses.

Other datasets from the FTA NTD are used in conjunction with the vehicle dataset such as the "2021 Agency Info" to extrapolate address information for the agencies. Some exploration of these data revealed that the NTD database contained information from some U.S. territories such as American Samoa, Guam, Puerto Rico, and the Virgin Islands. This information is considered to be outside of the scope of this analysis. By joining the agency data to the vehicle data, we filtered out 448 buses that did not operate in U.S states or D.C. and 3,604 that did not have an address, leaving 61,294 buses. Finally, for the purpose of being able to apply fuel emissions factors, we chose a subset of the data to analyze. This included the fuel types of diesel, CNG, gasoline, hybrid diesel, hybrid gasoline, and electric battery. These fuel types represent 93.0% of the fleet which means we are removing 4,283 vehicles from the data set and leaving 57,011 vehicles by only evaluating these fuel types. Diesel engines alone account for 69% of the total fleet. The rest of the fleet were mostly of unknown fuel types.

The "2021 Annual Database Service" dataset was used to sum the number of days the agencies were operating throughout the year. Some data exploration showed that there were three methods that agencies followed to report their service information. The first method was to report only annual service in summed totals. The second method was to report weekday and weekend services, and the last methods was to report by both methods. Larger agencies were generally operational the entire year, however, some smaller agencies tended to not operate as frequently. To

compensate for these different reporting techniques, each agency was checked for an annual service data point and if they did not have one then the weekday and weekend datapoints were summed together to create an annual service entry. For some smaller agencies that were not required to report their number of operational days, it was assumed that they operated 5 days a week for 52 weeks out of the year.

Finally, the “2021 Fuel and Energy” dataset of the NTD was used to gather the average efficiency of each fuel type per agency. Similar to the vehicle dataset, the energy data had to be filtered to only include bus data (Mode = MB). There were fields for questionable data in this dataset, but this was not considered. There were also separate miles driven, gallons of different fuel type used, and mpg per fuel type listed per agency. In case there were any agencies that listed miles and gallons but not mpg, mpg was recalculated for agencies that reported gallons of fuel greater than zero. Looking at the range of the fuel economy for the different fuel types revealed that there may have been some human error in data input. Some diesel and gasoline data were listed as greater than 100 mpg and some electric battery miles per kWh (mi/kWh) was listed as greater than 20 mi/kWh. All these observations were much greater than the averages across the U.S. and were considered outliers. A replacement of these values was made with the average fuel economy per fuel type. As mentioned, smaller agencies did not have to report all data to the FTA. In particular, they did not have to report fuel and energy data. There were 319 agencies that fell into this category and their fuel economies by fuel type were also estimated with the national averages.

There were a number of buses that were listed without a manufacturing year. This filter removed 93 buses leaving 56,918. The range of manufacture years of the buses is between 1948 to 2021, but only seven predated 1990. These buses seem to be used more as novelties, so they have also been removed from the dataset leaving 56,911 buses.

### *SI 1.3 Additional Figures*

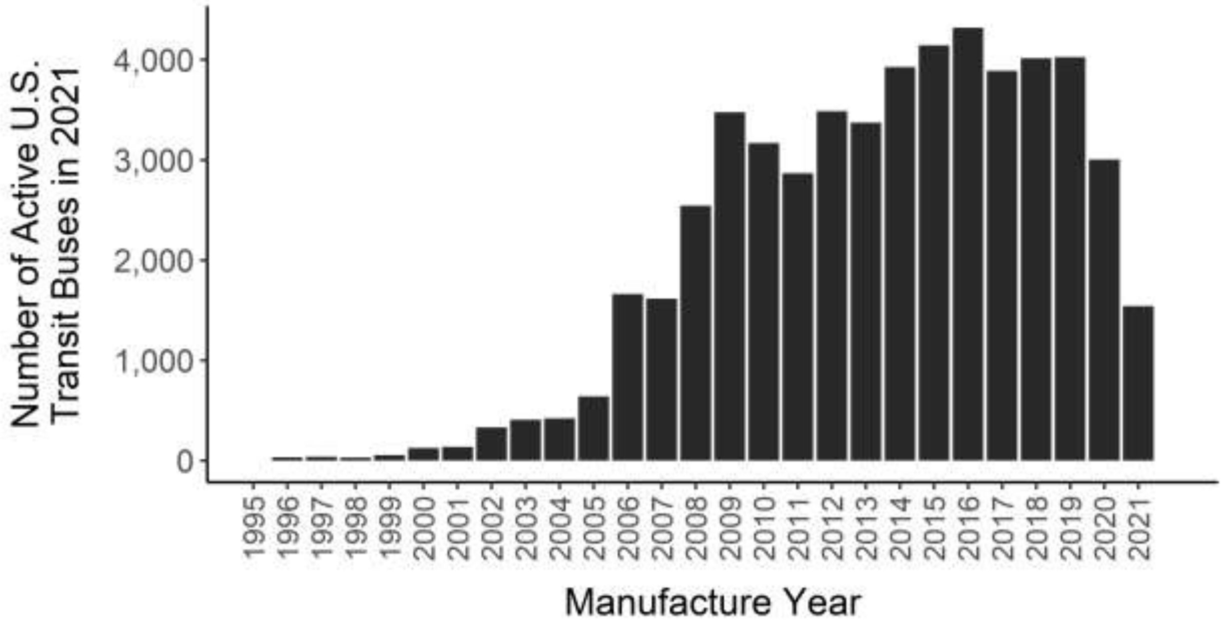


**Figure S1.** Distribution of manufacture year for the U.S. transit bus fleet operating in 2021

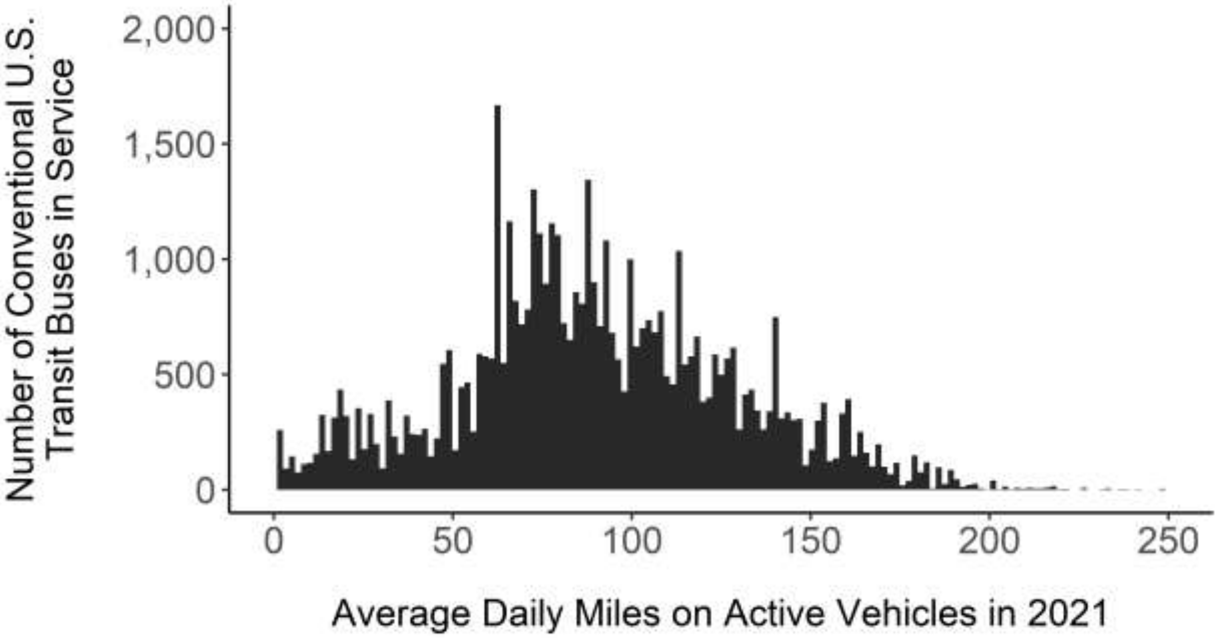


**Figure S2**. Average Daily Miles of Conventional U.S. Transit Buses in Active Service in 2021

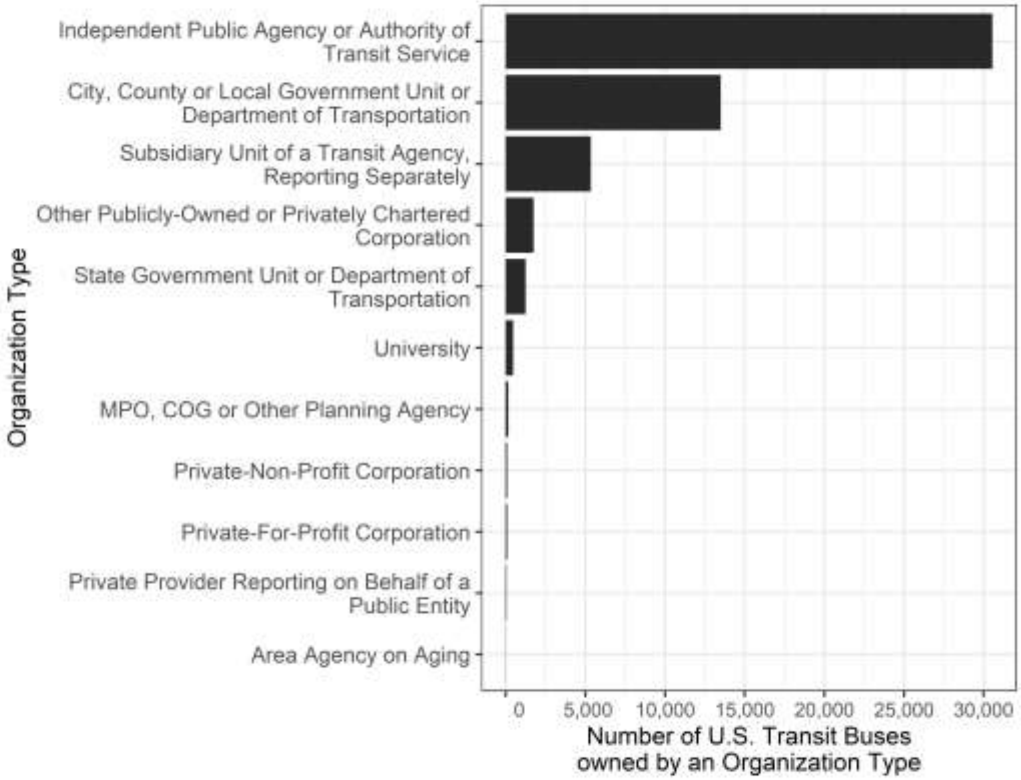


**Figure S3.** What types of organizations own the U.S. Transit Bus fleet?

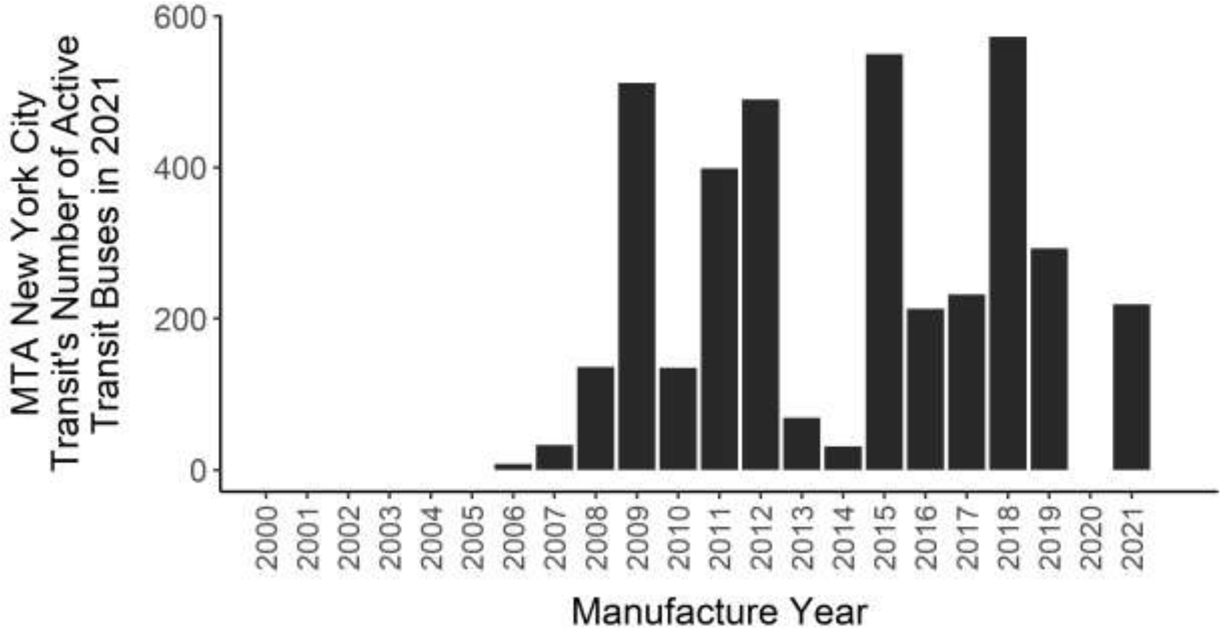


**Figure S4.** Distribution of manufacture year for MTA New York City Transit's active bus fleet in 2021.

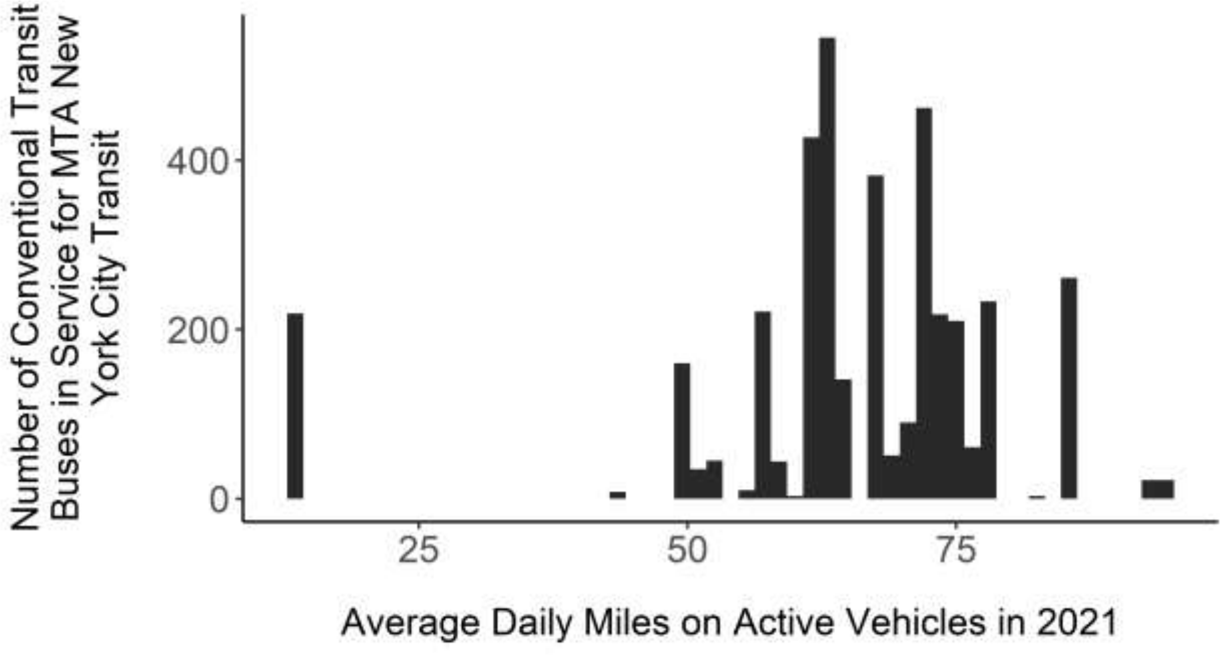


**Figure S5.** Average daily miles of conventional transit buses in active service in 2021in New York City's MTA

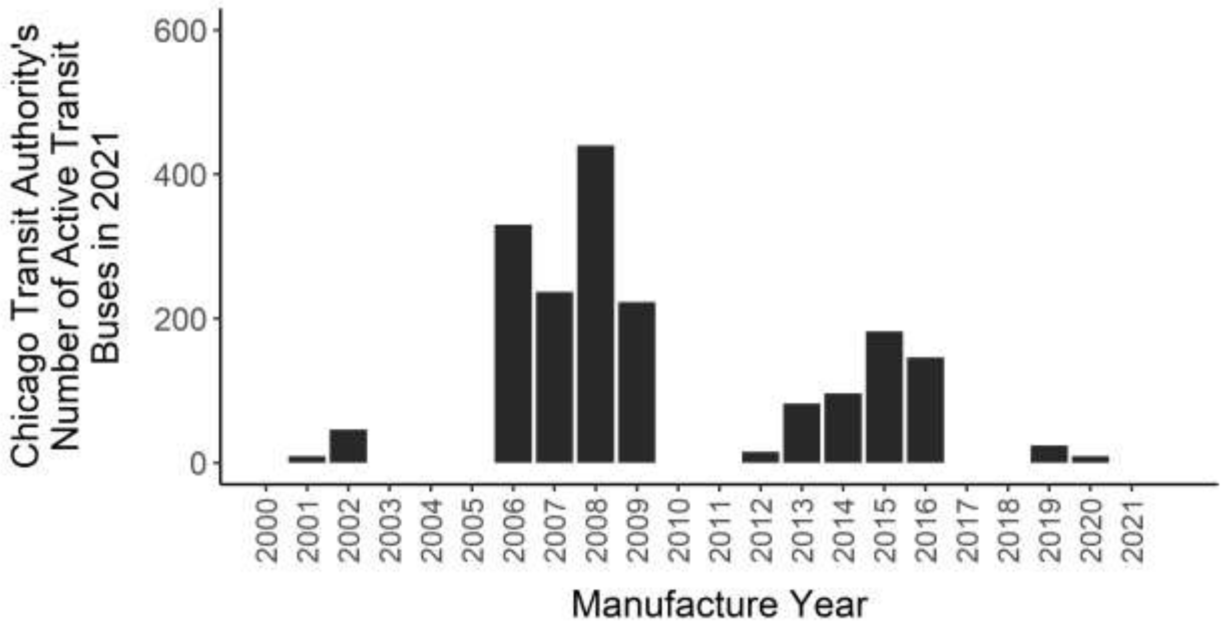


**Figure S6.** Distribution of manufacture year for Chicago Transit Authority's active bus fleet in 2021.

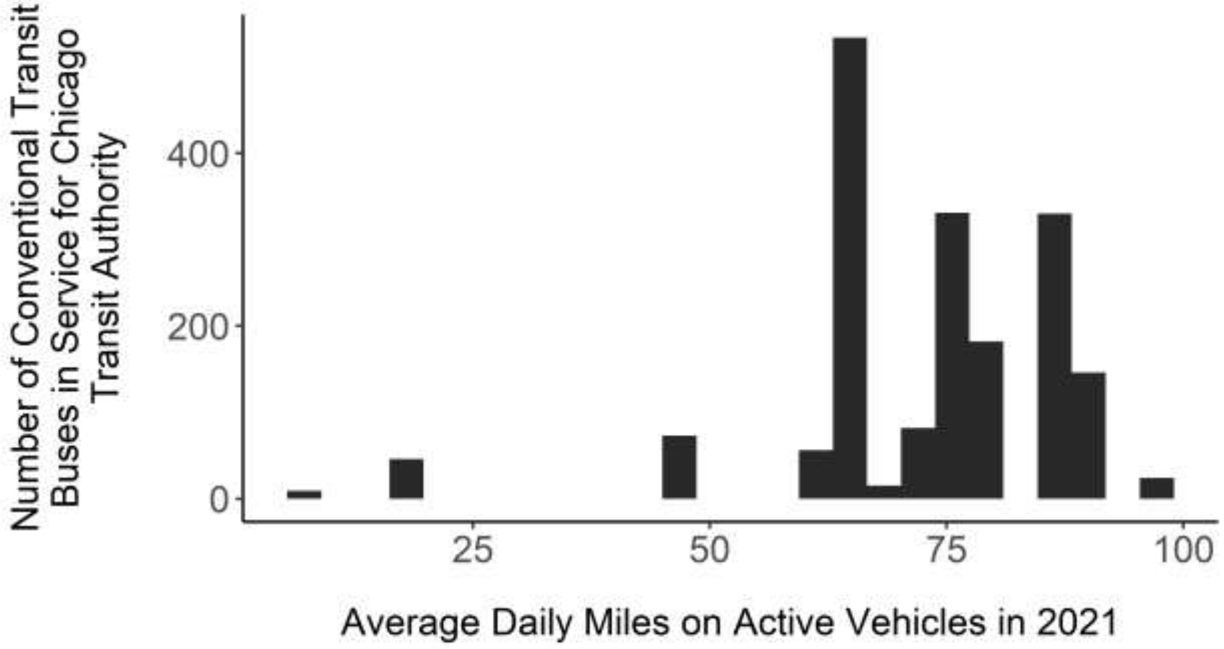


**Figure S7.** Average daily miles of Chicago Transit Authority conventional transit buses in active service in 2021

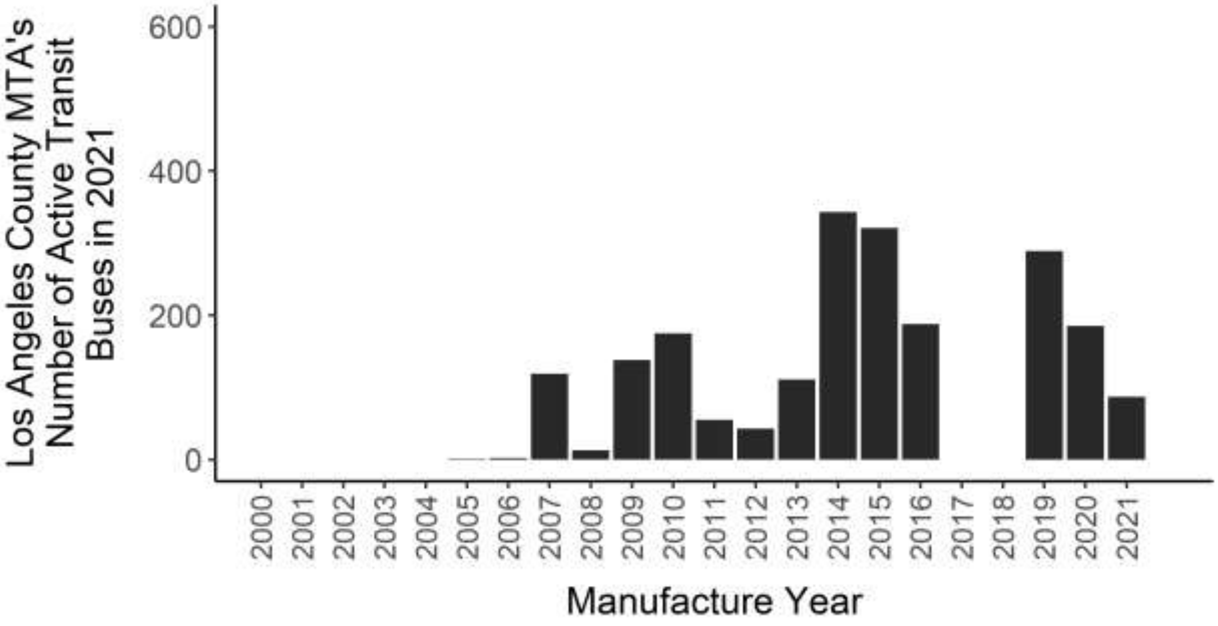


**Figure S8.** Distribution of manufacture year for Los Angeles County MTA's active bus fleet in 2021.

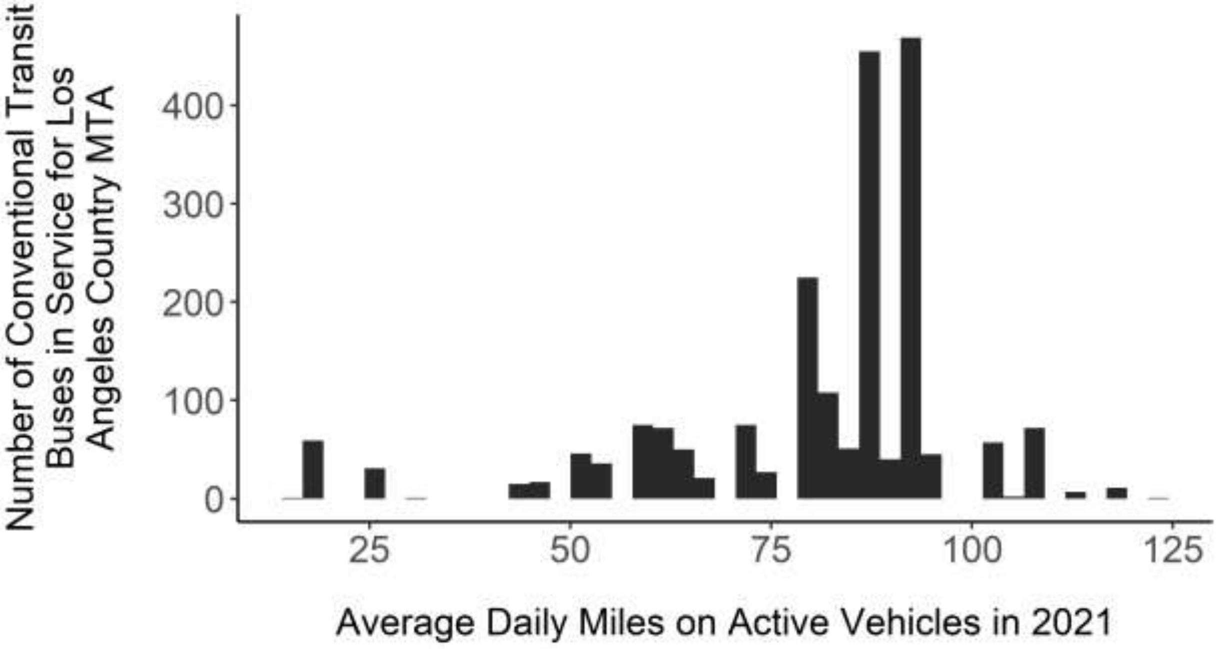


**Figure S9.** Average daily miles of conventional transit buses in active service in 2021in Los Angeles County MTA

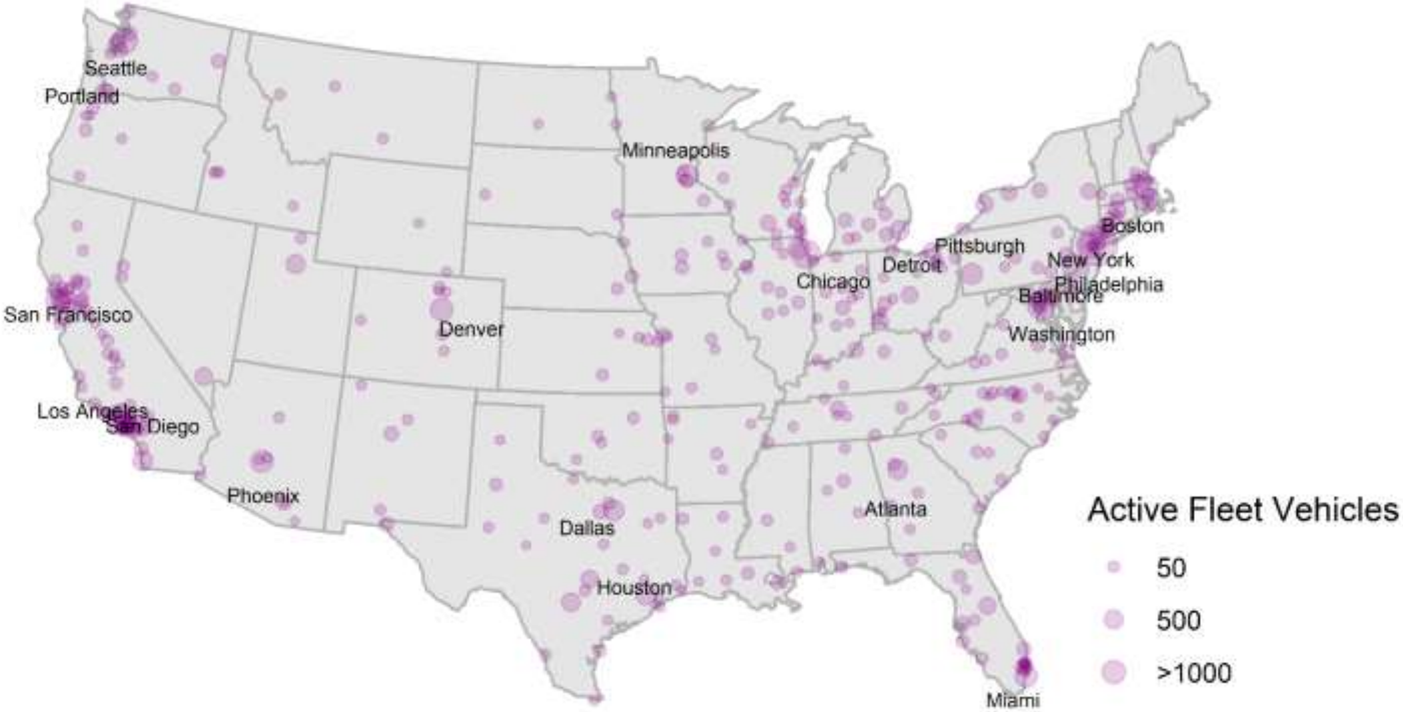


**Figure S10.** Map of U.S. transit bus fleets

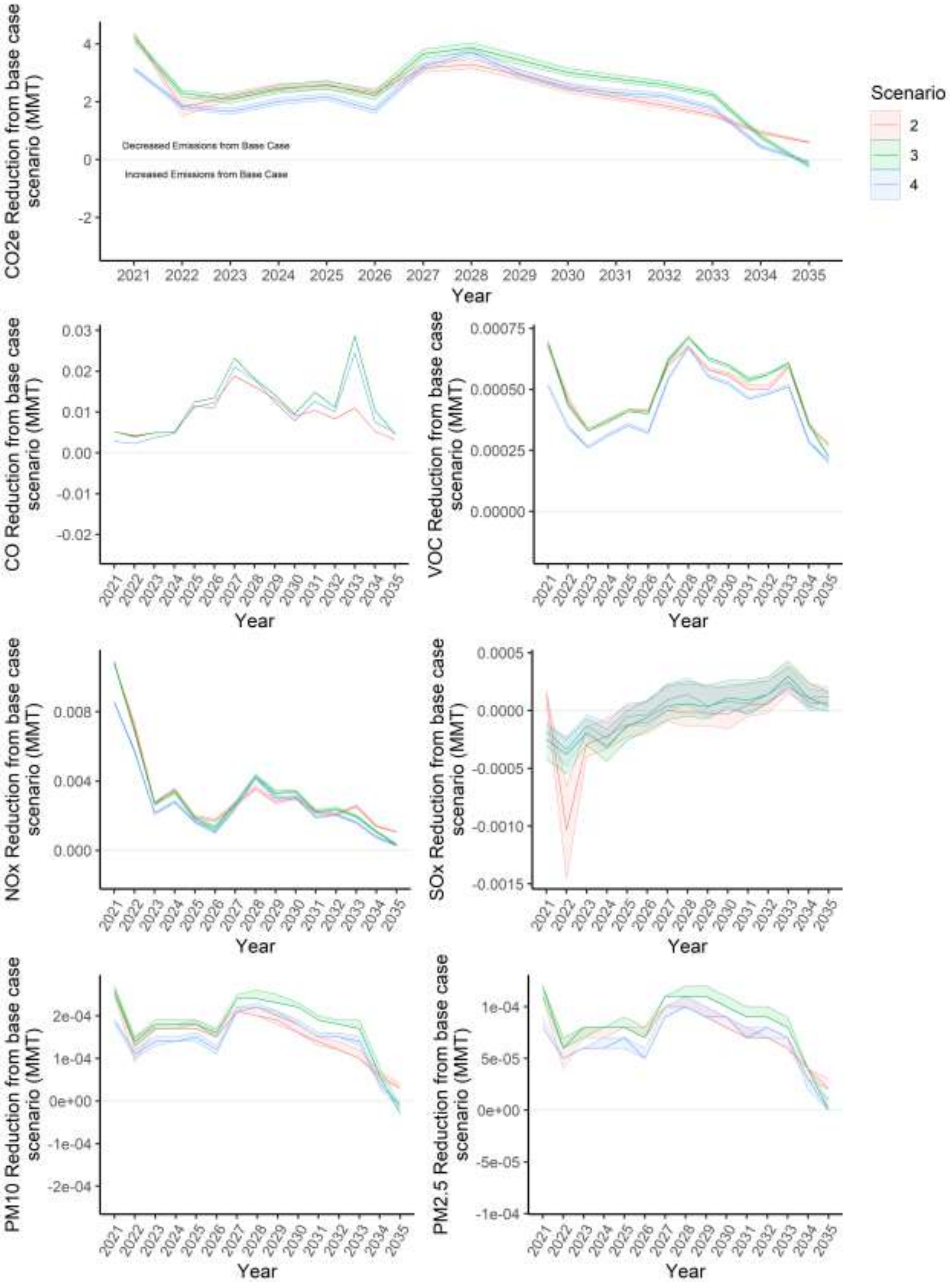


***Figure S11.*** *Yearly emission reductions in MMT from base case scenario to alternative scenarios. SOx is the only pollutant to increase. This is due to emissions associated with electricity generation*

### *SI 1.4 Methodology vs. AFLEET for vehicle emissions*

Another option we considered was using the Alternative Fuel Life-Cycle Environmental and Economic Transportation (AFLEET) Tool from the Argonne National Laboratory (2020). This tool also uses the GREET model [16] for the well-to-wheels fuel-cycle but uses the EPA's Motor Vehicle Emission Simulator (MOVES3) model [17] to estimate tailpipe emissions. The MOVES3 model has some drawbacks when looking at transit buses because they have no emission rate for the tire- and break-wear from BEBs. Also, their estimated total vehicle miles traveled (VMT) in 2019 were shown to be dramatically more than those reported in the FTA NTD. The raw value for VMT from the FTA was just over 2 million including the miles traveled from other non-transit bus type vehicles (such as school buses, minivans, SUVs and more). Whereas the MOVES3 data approximated almost 3.5 million VMT on just the diesel, gasoline, and CNG transit buses alone. While a portion of the AFLEET tool and our own model seek to quantify the emissions generated by the transit buses operated in the U.S., The AFLEET model in general makes broader assumptions. AFLEET uses the MOVES3 model from the EPA to get emission factors, while our analysis uses emissions from CARB. The emission values from CARB are measured directly from the tailpipe of some transit buses.

### *SI 1.5 EMFAC Completeness*

The EMFAC dataset was missing all tailpipe emission values for diesel-powered urban buses from 1990, 1992 to 1995 and 1997. All emissions were missing for compressed natural gas for the year 1998, and gasoline for 1999. Tire- and break-wear emission were missing for BEBs for the years 1996 and 1999. To fill in all these missing data points. Representative years were chosen for each fuel type to take place of the missing years. For example, the tailpipe emission for diesel in 1996 were used in place for all the missing years since these years only made up less than 0.3% of the diesel bus fleet. The CNG emission values from model year 1999 were used to replace those missing from model year 1998. This made up 0.07% of the CNG bus fleet. The gasoline values from model year 2000 were used to replace the missing values from model year 1999. This replacement affected less than 0.4% of the gasoline bus fleet. Finally, the tire- and break-wear emissions from model year 1999 were used in place of those from model year 1996 and 2001.

### *SI 1.6 Transition Methodology*

In all cases bus the base case, a replacement of the conventional U.S. transit bus fleet occurs with BEBs. To begin, the sensitivity of the model designates what consumption (kWh/mi) is used. In cases where the minimal emission scenarios are of interest, the "Low Consumption" value will be used for each bus model. These will then be multiplied by the average daily mileage the fleets drive. If this battery size is lower than the "Min Battery Size", then the buses will be assigned this minimum value. A similar procedure is used for any other value of Consumption (0-100%). In cases of the highest consumption, buses with calculated batteries sized larger than the "Max Battery Size" will be assigned this maximum value. See Table S1 below for the specifications used to inform these decisions. Furthermore, since the seat count of all our BEB models is less than 60, we double the proportion of the fleet that has seating capacity 60 or greater. This is roughly 1.5% of the U.S. fleet.

**Table S1.** BEB's identified from the NTD dataset that also had specification information online.

| Make Model | Chemistry | Low Battery Range (kWh) | High Battery Range (kWh) | Low Consumption (kWh/mi) | High Consumption (kWh/mi) | Lowest Range (mi) | Highest range (mi) | Seat Count |
|---|---|---|---|---|---|---|---|---|
| Byd Motors K7 | LFP | 180 | 215 | 1.15 | 1.84 | 68 | 131 | 23 |
| Byd Motors K9 | LFP | 324 | 350 | 1.91 | 2.64 | 86 | 128 | 38 |
| Byd Motors K11 | LFP | 578 | 652 | 2.09 | 3.74 | 108 | 218 | 56 |
| Gillig Corporation Low Floor Plus | NMC | 444 | 444 | 1.68 | 3.04 | 102 | 185 | 38 |
| New Flyer of America Xcelsior | NMC | 350 | 525 | 1.98 | 2.77 | 88 | 186 | 41 |
| Proterra Inc. Ecoride | LTO | 54 | 72 | 1.31 | 2.23 | 17 | 38 | 38 |
| Proterra Inc. Catalyst FC | LTO | 79 | 105 | 1.61 | 1.70 | 33 | 46 | 40 |
| Proterra Inc. Catalyst E2 | LTO | 440 | 660 | 1.53 | 2.28 | 135 | 302 | 40 |
| Proterra Inc. Catalyst Xr | NMC | 220 | 330 | 1.49 | 1.82 | 85 | 155 | 40 |
| Proterra Inc. Zx5 | NMC | 492 | 738 | 1.70 | 2.80 | 123 | 304 | 40 |

*SI 1.7 GHG and Air pollution emission scenarios sensitivity analysis*

**Table S2.** Range of input values for emission scenarios

| Sensitivity Parameters | Values for low emission estimate | Values for average emission estimate | Values for high emission estimates | Sources |
|---|---|---|---|---|
| Lifetime Battery Replacement | 0 | 1 | 1 | [18] |
| Battery Consumption Proportion Used | Min | Avg | Max | See SI 1.5 for more details |
| Depth of Discharge | 80% | 70% | 50% | [19] |
| EGrid Emission reductions from 2021 to 2035: | | | | [20] |
| $CO_2$ | -79% | -65% | -50% | |
| NOx | -82% | -62% | -42% | |
| SOx | -94% | -73% | -49% | |

*SI 1.8 LCOD Sensitivity Analysis*

This section provides more detail into the sensitivity of the LCOD analysis by varying each parameter individually to see the impact on the estimates and by providing an overall most optimistic and pessimistic value to show how low and high each value can reach with our assumptions.

**Table S3**. Optimistic and Pessimistic input values for levelized cost of driving sensitivity analysis

| | Fuel Type | | | |
|---|---|---|---|---|
| Parameter | Diesel | Compressed Natural Gas | Electric Rapid-Charge | References |
| Capital (2023$) | 728,000 | 897,000 | 952,000 | [21] |
| Infrastructure (%) | - | +10 | 68,000 | |
| | - | -10 | -10 | |
| Maintenance ($/mi) | 1.83 | 1.83 | 1.58 | [18] |
| | 0.40 | 0.40 | 0.19 | |
| Annual Vehicle Miles Traveled | 9,638 (Median VMT of BEB with test vehicles in fleet) | | | [14] |
| | 34,406 (Max VMT of NY MTA) | | | |
| Fuel Price ($/DGE) | 5.75 | 5.36 | 8.76 | [22], [23] |
| | 4.03 | 2.88 | 2.63 | |
| Discount Rate | 7 | | | |
| | 3 | | | |

The following table shows the results of this sensitivity analysis. Each value in each parameter and fuel type category is how the value LCOD value would vary given that one respective parameter changing to the values listed in **Table S3** above.

**Table S4.** Optimistic and Pessimistic results for levelized cost of driving sensitivity analysis ($/mi)

| | Fuel Type | | |
|---|---|---|---|
| Parameter | Diesel | Compressed Natural Gas | Electric Rapid-Charge |
| Capital | 5.48 (+18%) | 6.35 (+24%) | 5.86 (+21%) |
| Infrastructure | - | 5.16 (+0.6%) | 4.90 (+1%) |

| | | | |
|---|---|---|---|
| | - | 5.10 (-0.6%) | 4.65 (-4%) |
| Maintenance | 5.53 (+19%) | 6.02 (+17%) | 5.76 (+19%) |
| | 4.10 (-12%) | 4.59 (-11%) | 4.21 (-13%) |
| Annual Vehicle Miles Traveled | 8.43 (+82%) | 9.97 (+94%) | 10.29 (+113%) |
| | 3.74 (-19%) | 3.98 (-22%) | 3.44 (-29%) |
| Fuel Price | 4.89 (+5%) | 5.45 (+6%) | 5.05 (+4%) |
| | 4.53 (-2%) | 4.98 (-3%) | 4.56 (-6%) |
| Discount Rate | 4.96 (+7%) | 5.53 (+8%) | 5.29 (+9%) |
| | 4.34 (-6%) | 4.75 (-7%) | 4.27 (-20%) |

### *SI 1.9 Data Limitations*

Perhaps the largest source of uncertainty in the data used for this analysis is the use of the CARB EMFAC emission rates. These The CARB emissions only from transit buses in California. However, the emission rates were gathered from exhaust emission testing done by CARB, West Virginia University, and the FTA, so they have the potential to be applied more broadly (California Air Resources Board, 2021).

## SI 2 Access to Data

### *SI 2.1 FTA NTD Datasets*

The following is a list of the datasets used and mentioned in the main text:

- 2021 Revenue Vehicle Inventory [24]
- 2021 Annual Database Agency Information [25]
- 2021 Annual Database Service [26]
- 2021 Fuel and Energy [27]– The data used from this dataset was a single Excel sheet titled "Fuel and Energy". The other sheets were used for a data dictionary or for summarizations which were occasionally referred to but not processed in the code.

### *SI 2.2 Battery Cost Extrapolation Method*

The source of the battery costs from year to year were estimated using the Argonne National Laboratory's BEnefit ANalysis (BEAN) model (2021) for price points from the years 2021, 2027, and 2035 [28]. Using these price points and a decay equation, we were able to estimate battery pack prices for every year in between and slightly beyond. The equation used can be seen in Eq S1 below.

*Equation S1*

$$y = \frac{q-n}{b^p - b^m}(b^x - b^m) + n$$

To use this equation the constant "b" had to be estimated first. For medium, optimistic, and pessimistic technological progression, the years of 2021, 2027, and 2035 were all used as variables m, n, and x respectively. Their corresponding values for each technology progression were used as n, q, and y respectively. Then the equation was graphed, and b was estimated to the nearest thousandths place. Once the value of "b" was established for each tech progression, the values of p, q, m, and n were all used again while x was allowed to vary for each year and the y calculated was the estimated battery pack price for that year. The results can be seen in **Figure S12** below.

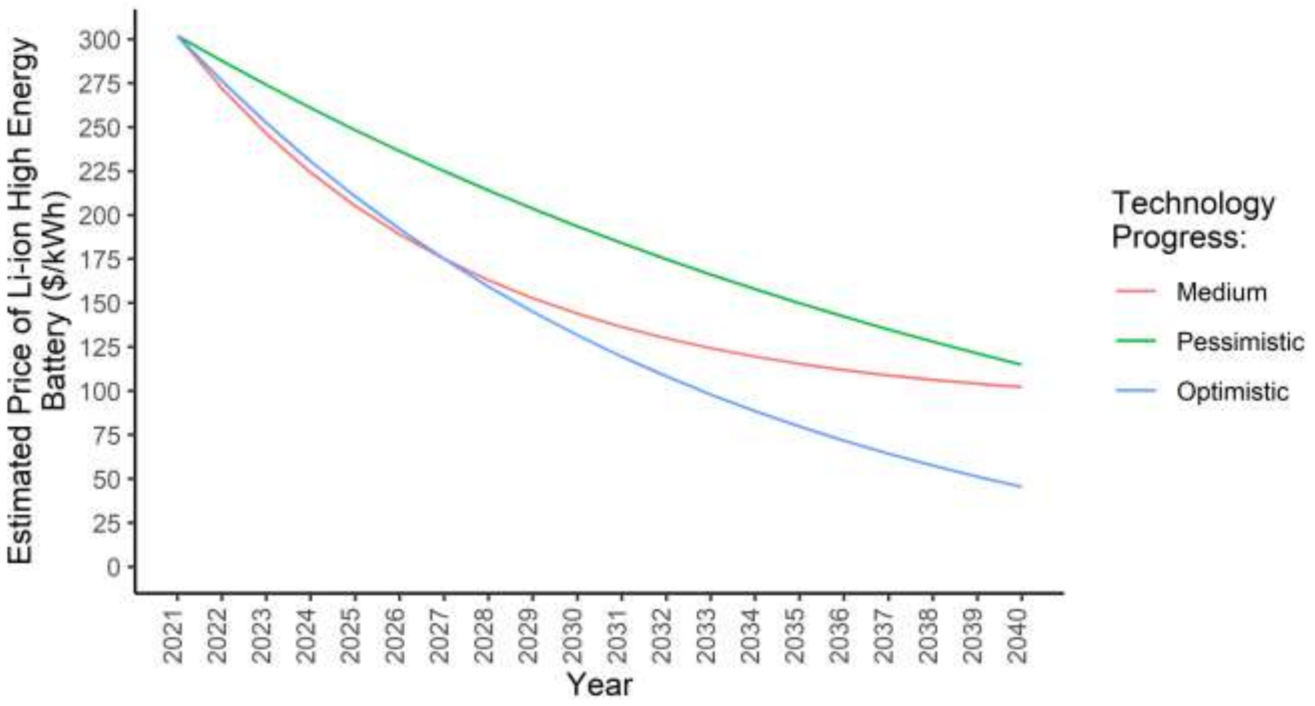


**Figure S12.** Forecasted Trend of Average Price of Li-ion High Energy Battery (Heavy-Duty Application) Depending on How Rapidly the Technology Progresses ($/kWh). Data from Argonne National Laboratory BEAN model.

**Table S5.** Fuel Specs from GREET Fuel Cycle [16]

| | Btu/gal | mmBtu/gal | $Ft^3$/DGE |
|---|---|---|---|
| Gasoline | 112,194 | 0.1121935 | |
| Low-sulfur diesel | 129,488 | 0.1294878 | |
| Natural gas (per cubic foot) | 983 | 0.0009830 | 143.94 |
| | | 1 kWh = 3412 Btu | |

*SI 2.3 Zenodo Data Access*

The following datasets and code for this project are openly available: https://doi.org/10.5281/zenodo.10613614

- EMFAC Emission Data [29]
- Petroleum Emissions from GREET Fuel Cycle [16]
- Natural Gas Emission from GREET Fuel Cycle [16]
- Battery Manufacturing Emissions from GREET Vehicle Cycle [16]
- eGRID Subregional Data [30]
- Zip Code Join File for Subregional eGRID Data [31]

**Supplemental Information References**


[1] S. M. Ahmed, J. P. Lemkau, N. Nealeigh, and B. Mann, "Barriers to healthcare access in a non-elderly urban poor American population," *Heal. Soc. Care Community*, vol. 9, no. 6, pp. 445–453, 2001, doi: 10.1046/j.1365-2524.2001.00318.x.

[2] S. T. Syed, B. S. Gerber, and L. K. Sharp, "Traveling towards disease: Transportation barriers to health care access," *J. Community Health*, vol. 38, no. 5, pp. 976–993, Oct. 2013, doi: 10.1007/S10900-013-9681-1.

[3] U.S. Energy Information Administration (EIA), "Frequently Asked Questions (FAQs)." [Online]. Available: https://www.eia.gov/tools/faqs/faq.php?id=74&t=11. (accessed: 2022-3-23)

[4] G. Schivley, I. Azevedo, and C. Samaras, "Assessing the evolution of power sector carbon intensity in the United States," *Environ. Res. Lett.*, vol. 13, no. 6, p. 064018, Jun. 2018, doi: 10.1088/1748-9326/aabe9d.

[5] S. Guo and E. Kontou, "Disparities and equity issues in electric vehicles rebate allocation," *Energy Policy*, vol. 154, p. 112291, Jul. 2021, doi: 10.1016/J.ENPOL.2021.112291.

[6] A. Aamodt, K. Cory, and K. Coney, "Electrifying Transit: A Guidebook for Implementing Battery Electric Buses," Apr. 2021. [Online]. Available: www.nrel.gov/publications.: (accessed: 2021-6-11)

[7] R. Nunno, "Fact Sheet: Battery Electric Buses: Benefits Outweigh Costs | White Papers," Environmental and Energy Study Institute. [Online]. Available: https://www.eesi.org/papers/view/fact-sheet-electric-buses-benefits-outweigh-costs. (accessed: 2020-9-27)

[8] N. Quarles, K. M. Kockelman, and M. Mohamed, "Costs and Benefits of Electrifying and Automating Bus Transit Fleets," *Sustainability*, vol. 12, no. 10, p. 3977, May 2020, doi: 10.3390/su12103977.

[9] U.S. Department of Transportation - Federal Transit Administration, "Low or No Emission Vehicle Program - 5339(c)." [Online]. Available: https://www.transit.dot.gov/funding/grants/lowno. (accessed: 2020-7-17)

[10] F. Tong, C. Hendrickson, A. Biehler, P. Jaramillo, and S. Seki, "Life cycle ownership cost and environmental externality of alternative fuel options for transit buses," *Transp. Res. Part D Transp. Environ.*, vol. 57, pp. 287–302, Dec. 2017, doi: 10.1016/j.trd.2017.09.023.

[11] A. Lajunen, "Energy consumption and cost-benefit analysis of hybrid and electric city buses," *Transp. Res. Part C Emerg. Technol.*, vol. 38, pp. 1–15, 2014, doi: 10.1016/j.trc.2013.10.008.

[12] S. P. Holland, E. T. Mansur, N. Z. Muller, and A. J. Yates, "The environmental benefits of transportation electrification: Urban buses," *Energy Policy*, vol. 148, p. 111921, Jan. 2021, doi: 10.1016/j.enpol.2020.111921.

[13] M. Mohamed, H. Farag, N. El-Taweel, and M. Ferguson, "Simulation of electric buses on a full transit network: Operational feasibility and grid impact analysis," *Electr. Power Syst. Res.*, vol. 142, pp. 163–175, 2017, doi: 10.1016/j.epsr.2016.09.032.

[14] Federal Transit Administration, "NTD Data." [Online]. Available: https://www.transit.dot.gov/ntd/ntd-data. (accessed: 2023-2-5)

[15] U.S. Department of Transportation - Federal Transit Administration, "National Transit Database 2018 Policy Manual for Reduced Reporters," 2018.

[16] "GREET Model," Argonne National Laboratory. [Online]. Available: https://greet.es.anl.gov/. (accessed: 2023-3-8)

[17] U.S. Environmental Protection Agency (EPA), "Latest Version of MOtor Vehicle Emission Simulator (MOVES)." [Online]. Available: https://www.epa.gov/moves/latest-version-motor-vehicle-emission-simulator-moves. (accessed: 2022-4-4)

[18] C. Johnson, E. Nobler, L. Eudy, and M. Jeffers, "Financial Analysis of Battery Electric Transit Buses," Golden, CO, Jun. 2020. [Online]. Available: https://www.nrel.gov/docs/fy20osti/74832.pdf: (accessed: 2022-3-16)

[19] A. Schabert, "How to avoid battery degradation in electric buses," *Automotive World*, Jun. 09, 2022. [Online]. Available: https://www.automotiveworld.com/articles/how-to-avoid-battery-degradation-in-electric-buses/: (accessed: 2023-8-5)

[20] J. Bistline *et al.*, "Emissions and energy impacts of the Inflation Reduction Act," *Science (80-. ).*, vol. 380, no. 6652, pp. 1324–1327, Jun. 2023, doi: 10.1126/SCIENCE.ADG3781.

[21] M. Dickens, "2020 Public Transportation Vehicle Database," Washington, DC, 2020. [Online]. Available: https://www.apta.com/research-technical-resources/transit-statistics/vehicle-database/

[22] U.S. Department of Energy, "Clean Cities Alternative Fuel Price Report, October 2021," Oct. 2021. [Online]. Available: https://afdc.energy.gov/files/u/publication/alternative_fuel_price_report_october_2021.pdf: (accessed: 2021-

12-19)
[23] U.S. Energy Information Administration (EIA), "Electric Power Monthly - December 2021 (Transportation))." [Online]. Available: https://www.eia.gov/electricity/monthly/epm_table_grapher.php?t=epmt_5_6_a. (accessed: 2021-3-4)
[24] Federal Transit Administration, "2021 Annual Database Revenue Vehicle Inventory," National Transit Database. [Online]. Available: https://www.transit.dot.gov/ntd/data-product/2021-annual-database-revenue-vehicle-inventory. (accessed: 2023-3-28)
[25] Federal Transit Administration, "2021 Annual Database Agency Information," National Transit Database. [Online]. Available: https://www.transit.dot.gov/ntd/data-product/2021-annual-database-agency-information. (accessed: 2023-3-28)
[26] Federal Transit Administration, "2021 Annual Database Service," National Transit Database. [Online]. Available: https://www.transit.dot.gov/ntd/data-product/2021-annual-database-service. (accessed: 2023-3-28)
[27] Federal Transit Adminstration, "2021 Fuel and Energy," National Transit Database. [Online]. Available: https://www.transit.dot.gov/ntd/data-product/2021-fuel-and-energy. (accessed: 2023-3-28)
[28] Argonne National Laboratory, "BEAN - Vehicle & Mobility Systems Group - Argonne National Laboratory." [Online]. Available: https://vms.es.anl.gov/tools/bean/. (accessed: 2022-3-16)
[29] California Air Resources Board, "EMFAC." [Online]. Available: https://arb.ca.gov/emfac/. (accessed: 2021-7-12)
[30] U.S. Environmental Protection Agency (EPA), "eGRID." [Online]. Available: https://www.epa.gov/egrid/download-data. (accessed: 2023-1-18)
[31] U.S. Environmental Protection Agency (EPA), "Power Profiler." [Online]. Available: https://www.epa.gov/egrid/power-profiler#/. (accessed: 2023-1-18)
